# Development of In Vitro Anther Culture for Doubled Haploid Plant Production in Indica Rice (*Oryza sativa* L.) Genotypes

**DOI:** 10.3390/plants12091774

**Published:** 2023-04-26

**Authors:** Csaba Lantos, Mihály Jancsó, Árpád Székely, Tímea Szalóki, Shoba Venkatanagappa, János Pauk

**Affiliations:** 1 Department of Biotechnology, Cereal Research Non-Profit Ltd., P.O. Box 391, H-6701 Szeged, Hungary; 2 Research Center for Irrigation and Water Management, Institute of Environmental Sciences, Hungarian University of Agriculture and Life Sciences, Anna-liget 35, H-5540 Szarvas, Hungary; 3 International Rice Research Institute, DAPO Box 7777, INGER & ASEAN RiceNet and NARVI Global Networks Rice Breeding Platform (S.V.), Metro Manila 1301, Philippines

**Keywords:** androgenesis, anther culture, flow cytometry, haploid, indica, *Oryza sativa* L.

## Abstract

Anther culture is an efficient biotechnological tool in modern plant breeding programs to produce new varieties and parental lines in hybrid seed productions. However, some bottlenecks—low induction rate, genotype dependency, albinism—restrict the widespread utilization of in vitro anther culture in rice breeding, especially in Oryza sativa ssp. indica (indica) genotypes, while an improved efficient protocol can shorten the process of breeding. Three different induction media (N_6_NDK_,_ N_6_NDZ, Ali-1) and four plant regeneration media (mMSNBK1, MSNBK3, MSNBKZ1, MSNBKZ2) were tested with five indica rice genotypes to increase the efficiency of in vitro androgenesis (number of calli and regenerated green plantlets). The production of calli was more efficient on the N_6_NDK medium with an average 88.26 calli/100 anthers and N_6_NDZ medium with an average of 103.88 calli/100 anthers as compared to Ali-1 with an average of 6.96 calli/100 anthers. The production of green plantlets was greater when calli was produced on N_6_NDK medium (2.15 green plantlets/100 anthers) compared when produced on to N_6_NDZ medium (1.18 green plantlets/100 anthers). Highest green plantlets production (4.7 green plantlets/100 anthers) was achieved when mMSNBK1 plant regeneration medium was used on calli produced utilizing N_6_NDK induction medium. In the best overall treatment (N_6_NDK induction medium and mMSNBK1 plant regeneration medium), four tested genotypes produced green plantlets. However, the genotype influenced the efficiency, and the green plantlets production ranged from 0.4 green plantlets/100 anthers to 8.4 green plantlets/100 anthers. The ploidy level of 106 acclimatized indica rice plantlets were characterized with flow cytometric analyses to calculate the percentage of spontaneous chromosome doubling. Altogether, 48 haploid-, 55 diploid-, 2 tetraploid- and 1 mixoploid plantlets were identified among the regenerant plantlets, and the spontaneous chromosome doubling percentage was 51.89%. Utilization of DH plants have been integrated as a routine method in the Hungarian rice breeding program. The tetraploid lines can be explored for their potential to offer new scopes for rice research and breeding directions in the future.

## 1. Introduction

Doubled haploid (DH) plant production methods have improved and led to accelerating the breeding of new varieties and hybrids. These methods are widely used in many crop plants such as barley, rapeseed, maize etc. Anther culture (AC) techniques can produce homozygous DH lines within one generation. Thus, the long process of conventional breeding methods can be reduced by homozygosity in early generations. The recessive alleles could be obtained and selected earlier due to the homozygosity of DH lines. The combination of AC with other plant biotechnological approaches (marker-assisted selection, in vitro selection, transgenic technology, genome editing) offers several opportunities for modern plant breeding programs. DH rice varieties were released via AC in several breeding programs [1,2,3,4,5]. However, some bottlenecks mitigate the widespread utilization of in vitro anther culture in rice breeding, especially in indica genotypes. Recently, several research groups have focused on the further improvement of AC in rice [6,7,8,9,10,11,12,13,14,15]. The first AC- derived green plantlet production was published in 1968 by Niizeki and Oono [16]; since then, significant improvements have been achieved by different research groups to improve the efficiency of AC [17,18,19,20]. Several factors such as genotype, growing conditions of donor plants, developmental stage of microspores, pre-treatments, and compositions of the induction and plant regeneration media influence the efficiency of in vitro AC. Furthermore, genotype dependency, low induction rate, plant regeneration rate and albinism limit the high frequency of doubled haploid (DH) plant production in rice, especially in indica genotypes [6,7,8,9,10,11,12,13,14,15].

Among *Oryza* species and subspecies, the efficiency of AC is significantly influenced by the genotype particularly among the indica genotypes, as lines and varieties are more recalcitrant in AC than in Oryza sativa ssp. japonica genotypes [6,13,21,22,23,24,25,26,27,28,29]. The necrosis of anthers leading to cell death, low frequency of callus induction, low plant regeneration efficiency and the high proportion of albinos are the most critical limiting factors for AC of indica genotypes [6,8,14,15,19,21].

The culture conditions of AC (induction medium, growth regulators, carbon source, temperature etc.) influence the efficiency of AC. In the indica rice, the most frequently applied basal medium was the N_6_ medium [13,30,31,32], but some other media (Ali-1, CIM) were also used for the induction of in vitro androgenesis in AC [28,29,33]. In the induction of androgenesis in rice, maltose is the most frequently applied carbohydrate source due to its positive effect on increasing callus induction and green plantlet regeneration [12,13,20,21]. Exogenous growth regulators, such as 1-naphtylacetic acid (NAA), 6-benzylaminopurin (BAP), 2,4-dichlorophenoxyacetic acid (2,4-D), kinetin, zeatin and their combinations, were applied for androgenesis induction of indica rice genotypes [13,23,28,29,30,32,33]. The development of microspore-derived calli was tracked from the initial cell division of uninucleate microspores until the callus formation in the indica rice genotype (At303) by Mayakaduwa and Silva [13].

According to published protocols, different combinations of growth regulators (BAP, NAA, kinetin and zeatin) were successfully applied to produce green plantlets and increase plant regeneration efficiency [5,7,8,10,11,12,13,14,21,22,23,24,26,27,30,31,34,35]. The efficiency of green plantlets production was improved further by the increased quantity of gelling agent and added proline to the regeneration medium in rice AC [30,36].

The number of green plants with diploid chromosome numbers (diploid) conferring fertility is one of the most important parameters in the DH plant production systems. Therefore, different methods (stomatal density, length and flow cytometric analyses) are developed for the identification of ploidy levels of regenerated plantlets [31]. The most accurate estimation can be conducted by flow cytometric analyses [31].

The aim of this study is to improve the androgenetic induction in AC of indica rice genotypes. In vitro androgenesis was induced in AC of five indica rice genotypes—Co 39, PSB RC 94, IR 64, IRRI 147 and Mangala—to study the response of different genotypes. These genotypes were provided by the INGER program of International Rice Research Institute based in the Philippines. The effects of different induction media N_6_NDK, N_6_NDZ and Ali-1were examined on the production of AC-derived calli and the efficiency of plant regeneration. In the plant regeneration phase, the combinations of different growth regulators were tested to increase the green plant regeneration efficiency. The percentage of the acclimatized green plantlets with different ploidy levels (haploid, diploid, tetraploid and mixoploid) was determined by flow cytometric analyses.

## 2. Results

### 2.1. Effect of Genotype, Induction Media and Their Interactions on Androgenesis Induction in AC

**Figure 1:**
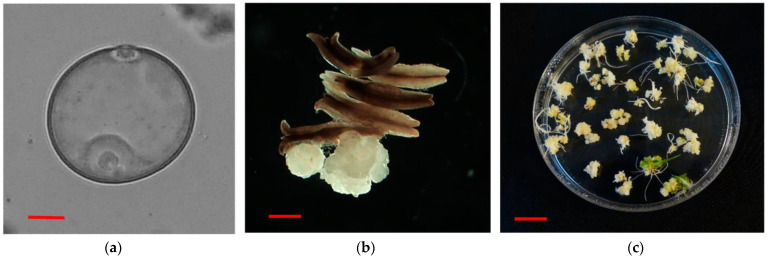
The in vitro AC of indica rice: (**a**) uninucleate microspore for androgenesis induction, (**b**) microspore–derived calli at 4-week-old AC and (**c**) plant regeneration of calli developed in AC. Red bars = 10 μm for (**a**), 1mm for (**b**) and 10 mm for (**c**).

Three different induction media were compared in in vitro AC of five indica rice genotypes, which were cultured at the uninucleate stage of microspores (Figure 1a). The microspore-derived calli were observed after 4 weeks of AC (Figure 1b), and later regenerated green and albino plantlets were seen on the regeneration medium (Figure 1c).

In vitro androgenesis induction was successful in AC of each genotype. Based on the statistical analysis (Table 1), the effect of genotype, the induction media and their interaction were shown to influence the number of calli significantly (*p* < 0.001).

Table 2 shows the calli/100 anthers and genotypic differences. The number of calli/100 anthers ranged from 0 to 214.4 depending on genotype and induction medium (Table 2). Among the three tested media, the lowest number of calli was seen on the Ali-1 induction medium, where only one (‘Co 39’) among the five tested genotypes produced calli (34.8 calli/100 anthers). The mean of AC-derived calli/100 anthers was 103.88 and 88.26 on the N_6_NDZ and N_6_NDK induction media, respectively. The genotype ‘Co 39’ was the most responsive based on the number of induced calli, but the calli production was also significant in ‘PSB RC 94’ and ‘Mangala’ genotypes. Altogether, the number of AC-derived calli was significantly higher on N_6_NDZ and N_6_NDK media than on the Ali-1 medium.

### 2.2. Plant Regeneration from AC-Derived Calli of Indica Genotypes

The AC-derived calli produced on the N_6_NDZ and N_6_NDK induction media were used in the plant regeneration experiments. The comparisons of the effects of four combinations of growth regulators were based on the number of regenerated plantlets are shown in Table 3. The Two-way ANOVA revealed that the genotype significantly (*p* < 0.001) influenced the number of regenerated plantlets (total, green and albinos) except the number of green plantlets regenerated from calli induced on N_6_NDK medium (Table 3). The effect of treatment (growth regulator combinations) was also significant except for the number of green plantlets regenerated from the N_6_NDZ-derived calli. The genotype × media interaction was significant in the case of the number of green plantlets regenerated from the N_6_NDZ-derived calli.

Data of plant regeneration experiments using the AC-derived calli induced on N_6_NDK medium has been shown in Table 4 and Table 5. The means of plant regeneration efficiency (total, green and albino) was highest using the mMSNBK1 medium. The highest plant regeneration efficiency (18.4 plantlets/100 anthers) was observed in AC of the ‘Co 39’ genotype. However, this genotype produced higher levels of albinos (18 albinos/100 anthers). The highest value of green plantlets production (8.4 green plantlets/100 anthers) was achieved in AC of ‘PSB RC 94’. Each of the four tested genotypes produced green plantlets in the best treatment, the green plantlets production ranged from 0.4 to 8.4 green plantlets/100 anthers depending on genotype.

Table 5 demonstrates the regeneration frequency of AC-derived callus induced on N_6_NDK medium. Altogether 4668 calli of the four tested genotypes were applied to compare the effect of four plant regeneration media. The highest plant regeneration frequency was achieved using mMSNBK1 medium and the percentage of green and albino plantlet regeneration were 4.03% and 6.34%, respectively. The percentage of green and albino plantlet regeneration was lowest on the MSNBKZ1 plant regeneration medium. ‘Co 39’ genotypes produced only two in vitro green plantlets, while the other genotypes (‘PSB RC 94’, ‘IRRI 147’ and ‘Mangala’) regenerated a few dozen green plantlets.

Table 6 and Table 7 show the data of the plant regeneration experiments using the AC-derived calli induced on the N_6_NDZ medium. The means of total, albino and green plant regeneration efficiency were 9.08, 7.9 and 1.18 plantlets/100 anthers, respectively. The regenerated plantlets were mostly albinos in this experiment. In the best treatment, the highest mean of green plantlet regeneration (2.72 green plantlets/100 anthers) was achieved using the MSNBK3 medium containing 0.5 mg/L, 0.5 mg/L and 1.5 mg/L NAA, BAP and kinetin, respectively. However, the means of green plantlets production were significantly lower than in the first experiment. In this treatment, the green plantlets production of ‘Mangala’ was outstanding as an exception. Green plantlets production was successful from the AC-derived calli of only two tested genotypes.

Table 7 shows the efficiency of green and albino plant regeneration of AC-derived calli induced on the N_6_NDZ induction medium. Altogether 5276 calli of the five tested genotypes were placed on the four plant regeneration media to compare their effect on the number of green and albino plantlets. The green plantlet regeneration efficiency was highest using the MSNBK3 plant regeneration medium and the percentage of green plant regeneration was 2.58%. However, this value was lower than the green plantlets production of mMSNBK induction medium (4.03%) and calli produced on N_6_NDK induction medium (Table 5). The percentage of green plant regeneration was lowest on the MSNBKZ2 plant regeneration medium (0.3%).

‘Mangala’ genotypes produced 53 in vitro green plantlets in the experiment, while ‘PSB RC 94’ and ‘IRRI 64’ regenerated only a few green plantlets. The ‘Co 39’ and ‘IRRI 147’ genotypes regenerated only albino plantlets from the calli induced on the N_6_NDZ induction medium.

The regenerated green plantlets were grown in individual tubes and later jars till the transplantation of well-developed green plantlets into the greenhouse. The AC-derived green plants acclimatized to the greenhouse conditions well.

### 2.3. Spontaneous Chromosome Doubling of AC-Derived Green Plantlets as Demonstrated by the Ploidy Levels Using Flow Cytometric Analysis

**Figure 2:**
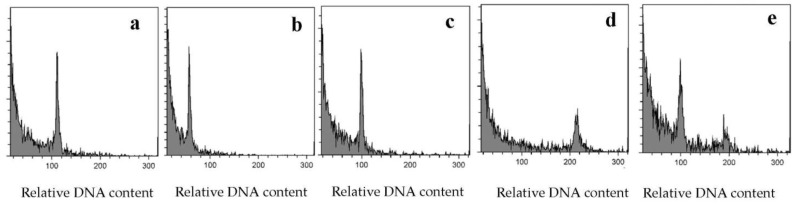
Flow cytometric analyses of AC-derived rice plantlets indica: histograms demonstrate the relative DNA content of leaves samples of (**a**) control, (**b**) haploid (**c**) diploid (**d**) tetraploid and (**e**) mixoploid acclimatized plantlet.

The ploidy levels of the AC-derived plantlets of five genotypes were analyzed by flow cytometric analyses. Figure 2 shows the relative DNA content of the five genotypes used in this study.

Altogether, 48 haploids (Figure 2b), 55 diploids (Figure 2c), 2 tetraploids (Figure 2d) and one mixoploid (Figure 2e) plantlets were identified among the acclimatized plantlets based on the histograms of flow cytometric analyses.

Table 8 demonstrates the ploidy level of the acclimatized plantlets by genotype. The percentage of spontaneous chromosome doubling was 51.89% among the acclimatized plantlets. Altogether, the seeds of 55 fertile DH lines were harvested, and two partially fertile tetraploid lines produced a few seeds. The produced DH lines of ‘Mangala’, ‘IRRI 147’ and ‘PSB RC 94’ genotypes were 27, 15 and 13 DHs, respectively.

**Figure 3:**
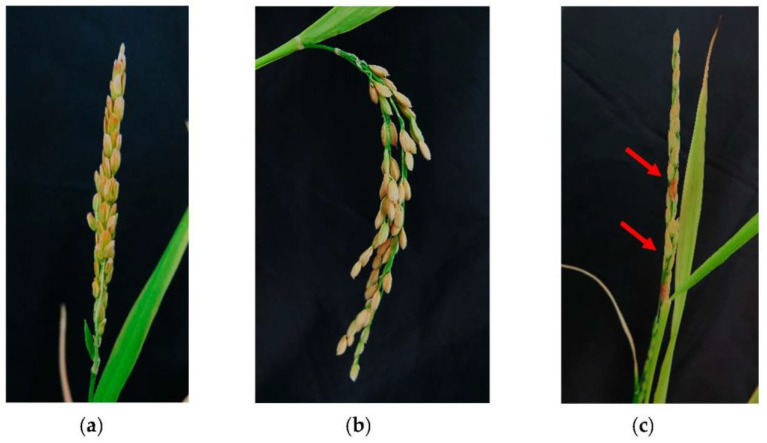
Fertility of AC—derived haploid (**a**), diploid (**b**) and tetraploid (**c**) plantlets: (**a**) sterile, (**b**) fertile, (**c**), partially fertile panicles (red arrows show the fertile seeds in panicle).

The fertility of the DH_0_ plantlets tested by flow cytometric analyses was checked based on seed production (Figure 3). The haploid plants produced panicles without any seed (sterile), the diploid plantlets were fertile, while the panicles of tetraploid plants contained a few seeds (partially fertile).

## 3. Discussion

### 3.1. Induction of Androgenesis in Indica Rice

The N_6_ basal medium is the most generally used medium for the induction of rice androgenesis [13,30,31,32], while Ali et al. reported the superiority of the Ali-1 induction medium compared to N_6_ medium in AC of rice [28]. The present study compared the N_6_NDK induction medium to Ali-1 and N_6_ basal medium supplemented with combinations of different growth regulators (N_6_NDZ). In contrast to the report by Ali et al. [28], the induction media prepared with N_6_ basal medium (N_6_NDK and N_6_NDZ) outperformed the Ali-1 induction medium with regard to callus production. The number of produced calli was limited on Ali-1 induction medium [28]. N_6_NDK medium proved an efficient induction medium regarding callus production in AC of in indica rice genotypes.

### 3.2. Effect of Induction Medium on Plant Regeneration

The regeneration of green plantlets is the next critical point of DH plant production in indica rice genotypes because of the low efficiency of plant regeneration and the high number of albinos [6,8,14,15,19,21,22,25,30]. The induction medium affects the regeneration ability of calli [23]. There were differences among the plants regenerated utilizing the generated on the best two induction media (N_6_NDK and N_6_NDZ) with differences in the combination of growth regulators. The number of total regenerated plantlets was 6.406 plantlets/100 anthers and 9.08 plantlets/100 anthers from calli produced on N_6_NDK and N_6_NDZ induction media, respectively.

The number of albinos from calli induced on N_6_NDZ was 7.9 albinos/100 anthers while the number of green plantlets was 1.18 green plantlets/100 anthers on average. The calli produced on N_6_NDK induction medium regenerated 4.256 albino/100 anthers and 2.15 green plantlets/100 anthers on average. The number of regenerated green plantlets was almost two times higher using the N_6_NDK induction medium, indicating that this induction medium is the best medium to be utilized to ensure sufficient green plant generation. Zeatin in combination with 2,4-D and NAA was applied for androgenesis induction in rice [28]. Although the number of produced calli was higher using this combination of growth regulators, adverse effects of this induction media (N_6_NDZ) were observed on plant regeneration. Interestingly, the N_6_NDK medium was efficient in the induction of in vitro androgenesis of japonica genotypes as well [37].

The percentage of green plantlet regeneration of callus was also higher from callus induced on N_6_NDK (on average 1.84%) than N_6_NDZ (on average 1.1%), these values ranged from 0% to 47.06%, which were influenced by genotype and plant regeneration medium. According to some relevant publications, green plantlet production ranged from 0% to 58.25% depending on genotype and culture conditions in indica rice AC [13,29,30,31,32].

### 3.3. Effect of Exogenous Growth Regulators on the Regeneration of Green Plantlets

The compositions of plant regeneration medium influence the efficiency of green plantlets production in AC of rice. The MS basal media have been frequently applied for plant regeneration in rice AC [8,11,12,38]. Sucrose is a general carbon source during plant regeneration, while a wide range of growth regulators (BAP, IAA, NAA, kinetin and zeatin), and their combinations were applied for plant regeneration of AC-derived rice calli [11,12,21,23,24,26,28,38].

In the plant regeneration experiments, the genotypes, combination of growth regulators and quality of calli (see above) significantly influenced the plant regeneration efficiency. Zeatin was applied as an exogenous growth regulator in combination with other hormones in rice AC experiments [26,28,35], while added zeatin had an adverse effect on both callus induction and plant regeneration in AC of indica rice genotypes. The different combinations of NAA, BAP and Kinetin were applied for plant regeneration in rice AC [11,12,30]. Rout et al. reported that the combination of 0.5 NAA, 1.5 BAP and 0.5 Kinetin was efficient for green plantlets production in AC of indica rice hybrid [11]. However, the combination of 1 mg/L NAA, 1 mg/L BAP and 1 mg/L Kinetin was more efficient for green plant production than the combination of 0.5 mg/L NAA, 1.5 mg/L BAP and 0.5 mg/L Kinetin in this study. The green plantlet production was highest (4.7 green plantlets/100 anthers, average of genotypes) using calli derived from the N_6_NDK induction medium and mMSNBK1 plant regeneration medium containing 1 mg/L NAA:1 mg/L BAP:1 mg/L Kinetin; the number of regenerated green plantlets ranged from 0.4 to 8.4 green plantlets/100 anthers depending on genotype. In the treatment, the percentage of green plantlets regeneration of calli was on average 4.03%, ranging from 0.21% to 47.06% depending on genotype. The green plantlet productions are influenced by genotype (ranging from 0% to 58.25%) in indica rice AC [13,29,30,31,32], and higher efficiency of AC was reported in indica rice hybrids [29,30,32].

This indicated that this medium and growth regulator combination was probably the best combination suited for DH green plant production for rice plant breeding as per this study. It should be noted that these media and growth regulator combinations were also effective for green plantlets production in AC of japonica genotypes [37].

### 3.4. Identification of the Ploidy Level of AC-Derived Plantlets by Flow Cytometric Analyses

Flow cytometric analyses, the most accurate method to measure the ploidy levels of the microspore-derived plantlets, were used on 106 AC-derived acclimatized plantlets generated in this study. Overall, 48 haploids, 55 diploids, 2 tetraploids and a single mixoploid plantlet were identified among them. Based on these data, the spontaneous chromosome doubling was 51.89% average across genotypes. A significant difference was observed among the tested genotypes because of the different numbers of regenerated plantlets per genotype and, in turn, genotype dependency of chromosome doubling. Results recorded in this study were similar results (42.24–69.00%) to those recorded in most studies [2,5,6,21,24,37], but Cha-um et al. [27] observed lower values of spontaneous chromosome doubling (21.5–31.9%).

Since the performance of DH lines should be uniform in the following generations, stability of the ploidy of DH is also critical in plant breeding. as in some cases green plants can result from anther walls instead of the microspores. Hence it is critical that the uniformity of the regenerated diploid plants can be checked in the nursery in the DH_1_ and DH_2_ generations.

Two tetraploid plantlets (1.89%) with partially fertile panicles were identified among acclimatized plantlets in this study. Mishra et al. [32] reported a range of 6.3–16.6% in their study. The irregular development of pollen grains was mentioned among the causes of incomplete fertility [32].

It should be noted that the 48 haploids after the confirmation using flow cytometric analyses, the chromosome number of haploids can be doubled by chemical treatment (colchicine), which is feasible before (in vitro) [14,15] or after acclimatization (in vivo) [2]. This will further improve the efficiency of green DH plant regeneration in rice. Nevertheless, tetraploid rice plants are of interest in polyploid research and breeding programs [39].

## 4. Materials and Methods

### 4.1. Plant Materials and Growing Conditions

In the experiments, five indica rice genotypes (‘Co 39’, ‘PSB RC 94’, ‘IR 64’, ‘IRRI 147’ and ‘Mangala’) were tested in AC. These genotypes were provided under the International Genetic Evaluation of Rice (INGER) program based at International Rice Research Institute (INGER), a CGIAR member center based in the Philippines. The donor plants were grown at the Rice Research Station of the Hungarian University of Agriculture and Life Sciences (MATE), Institute of Environmental Sciences (IES), Research Center for Irrigation and Water Management (ÖVKI), Szarvas, Hungary. The standard rice cultivation method was used to grow donor plants in the nursery. After direct seeding, pre-emergent herbicide spraying (active ingredient: pendimethalin) was applied. After 5 weeks of seeding, permanent flooding was applied with a 5–20 cm irrigation level depending on the development of the plants. Furthermore, 60–60 kg/ha (total amount of 120 kg N/ha) of nitrogen fertilizer was applied two times (38 and 70 days after sowing). During the plant growing season, weeds were controlled manually.

### 4.2. Collection and Pre-Treatment of Donor Tillers

The tillers of donor genotypes were harvested at the booting stage when the panicles were covered with flag leaves and the distance was 2–5 cm between the flag leave and the second leaf. The donor tillers were placed into an Erlenmeyer flask containing tap water and were wrapped with PVC bags. The cold pre-treatment was carried out at 10 °C for three to seven days.

### 4.3. Sterile Technic and Isolation of Anthers

After pre-treatment, the developmental stages of microspores were identified by evaluation with Olympus CK-2 inverted microscope (Olympus Ltd., Southend-on-Sea, UK). The florets of panicles containing early- and mid-uninucleate microspores were surface-sterilized for 20 min in 2% sodium hypochlorite solution with 2 drops of Tween-80. After sterilization, the donor materials were rinsed three times with sterile distilled water. The anthers (100/Petri dish) were isolated from the donor florets and placed in the 60 mm diameter plastic Petri dishes which contained different induction media. At least 3000 anthers were isolated from each genotype to test the efficiency in vitro AC with three induction media and five genotypes.

### 4.4. Condition of Anther Culture for Induction of Calli

The effects of three different induction media were tested on the number of induced calli and regenerated plantlets in indica rice AC (Table 9). These media were Ali-1 induction medium [28], N_6_NDZ and N_6_NDK. For the N_6,_ media combinations, the N_6_ basal medium [40] was supplemented with two different combinations of growth regulators. They were induction media named here as N_6_NDZ (1 mg/L NAA, 1 mg/L 2,4-D and 0.1 mg/L Zeatin), and N_6_NDK (2.5 mg/L NAA, 1 mg/L 2,4-D and 0.5 mg/L kinetin). Both media contained 60 g maltose, 500 mg/L L-Proline and 500 mg/L L-Glutamine, pH was adjusted at 5.8. The medium was solidified with 2.8 g/L Phytagel. Furthermore, the Petri dishes with anthers cultured were incubated at 28 °C in a dark thermostat.

### 4.5. Plant Regeneration

The collections of AC-derived calli were finished on the 8th week of cultivation. The AC-derived calli were transferred to the 90 mm diameter plastic Petri dishes containing plant regeneration media. In the plant regeneration experiment, the AC-derived calli produced on the N_6_NDZ and N_6_NDK media were used to compare the effect of four different combinations of growth regulators on plant regeneration efficiency (green and albino plantlets).

During the plant regeneration period, the effects of different combinations of growth regulators (NAA, BAP, Kinetin and Zeatin) were tested on the regeneration of green and albino plantlets. The MS [41] medium was supplemented with growth regulator combinations, 30 g/L sucrose and pH was adjusted at 5.8 (Table 10). The plant regeneration media were solidified with an increased quantity (4 g/L) of Phytagel compared to earlier published MSNKB1 plant regeneration medium [37].

The plantlets with shoots were transferred into the glass tubes containing ½MS medium supplemented with 30 g/L sucrose, pH was adjusted at 5.8 [37]. The rooting medium was solidified with 2.8 g/L Phytagel. The green plantlets were cultured in glass tubes till the end of rooting. The well-rooted and tillered green plantlets were transplanted into 720 mL glass jars containing the same ½MS medium till transplantation of plantlets to the greenhouse. During the plant regeneration period, 16 h artificial light and constant temperature (24 °C) were kept in a tissue culture growing chamber.

### 4.6. Acclimatization of Plantlets

In the first half of March, the well-rooted green plantlets were transferred to the greenhouse. The green plants were transplanted individually into plastic pots (volume 1000 mL) containing 1:1:1 sand, peat and humus mixture, and the plantlets were covered with PVC bags to retain high humidity. After the acclimatization period (5 days), the PVC bags were removed and the acclimatized green plants were grown till harvest in the greenhouse. Ploidy levels of regenerated plants were checked by flow cytometric analyses and seed production.

### 4.7. Flow Cytometric Analyses

The ploidy levels of AC-derived plants and seed-derived control plants were determined by flow cytometric analysis using CytoFLEX Flow Cytometer (Beckman Coulter International S.A., Nyon, Switzerland). The samples (100 mg/plant) of young leaves were collected from the acclimatized plantlets grown in the greenhouse. The samples were destroyed in Eppendorf tubes containing 1 mL Galbraith buffer and two stainless steel beads using TissueLyser II (Qiagen, Hilden, Germany) at 20 Hz for 2 min to isolate the nuclei from the young leaves of plantlets [42]. The suspensions were purified using 20 μm sieves and 10 μL RNase solution was added to each sample at room temperature for 60 min to degrade the RNA content in the samples. DNA content was painted with 40 μL Propidium Iodide (PI) solution (1 mg/L) for 30 min at room temperature. After the preparation, the DNA content of samples was measured by flow cytometer, and the ploidy levels of samples were determined based on histograms.

### 4.8. Statistical Analyses

The experiments were carried out with at least ten replications (ten AC/induction medium/genotype). Data of AC response (number of calli, regenerated plantlets, albino- and green plantlets) were analyzed by 2-way ANOVA. The statistical analyses were carried out by Microsoft^®^ excel 2019 software developed by Microsoft (Redmond, WA, USA).

## 5. Conclusions

DH plant production methods are widely used in crop breeding and research programs because of their ability to produce genetically pure lines in one generation [3,43,44,45,46,47,48]. In vitro androgenesis was induced in AC of indica rice genotypes. Significant green plantlets production was achieved in the best treatment (4.7 green plantlets/100 anthers on average) using N_6_NBK induction medium and mMSNBK plant regeneration medium, while an adverse effect of exogenous zeatin (induction and plant regeneration media) was observed on the number of regenerated green plantlets. The methodological improvements mitigate the genotype dependency of AC step by step to enhance the practical utilization of the method. The mean spontaneous chromosome doubling was 51.89% based on flow cytometric analyses. Although the genotype influenced significantly the efficiency of AC, this method can be used for DH plant production in indica genotypes and their F_1_ combinations. Utilization of DH rice plants with genes for climate resilience will be highly useful to reduce the cycle of breeding time and adding new effective genetic combinations for climate resilience while maintaining superior yield and pest and disease tolerances for mitigation and management of climate change.

## Figures and Tables

**Table 1:** Statistical analyses (Two-way ANOVA) of the effect of induction media and genotype on the number of AC-derived calli of indica rice genotypes.

	df	MS of Calli/100 Anthers
Genotype	4	88,354.33 ***
Induction Media	2	135,393.00 ***
Interaction	8	23,128.62 ***
Error	135	6070.848

df: Degrees of Freedom. MS = Mean Square. *** significance at *p* < 0.001.

**Table 2:** The number of AC-derived calli/100 anthers for genotypes on different induction media and genotypes.

	Induction Media		
Genotype	N_6_NDK	N_6_NDZ	Ali-1
‘Co 39’	163.30 b A	214.40 a A	34.80 c A
‘PSB RC 94’	166.40 a A	83.80 b C	0.00 c A
‘IR 64’	0.00 a C	26.80 a D	0.00 a A
‘IRRI 147’	13.6 a C	30.00 a D	0.00 a A
‘Mangala’	98.00 b B	164.40 a B	0.00 c A
Mean	88.26	103.88	6.96

Note: Values followed by the same letters (a, b, c) are not significantly (*p* < 0.05) different for different media within the genotype. Values followed by the same capital letters (A, B, C, D) are not significantly (*p* < 0.05) different for the genotypes using the same medium.

**Table 3:** Statistical analyses (Two-way ANOVA) of overall plant regeneration efficiency (number of green and albino plantlets) for AC-derived calli induced on N_6_NDK and N_6_NDZ induction media on different plant regeneration media with different growth regulator combinations.

Induction Media		N_6_NDK			N_6_NDZ		
	df	MS of Green Plantlets	MS of Albinos	MS of Reg. Plantlets	MS of Green Plantlets	MS of Albinos	MS of Reg. Plantlets
Genotype	4	89.20 ns	829.97 ***	513.47 **	223.68 ***	1245.80 ***	2096.08 ***
Growth reg.	3	166.80 *	374.39 ***	1012.49 ***	57.04 ns	1397.20 ***	1834.77 ***
Interaction	12	36.93 ns	81.95 ns	78.40 ns	52.37 **	159.67 ns	287.44 ns
Error	180	46.33	46.51	113.73	22.73	153.57	202.65

df = Degrees of Freedom. MS = Mean Square. *** significant at *p* < 0.001. ** significant at *p* < 0.01. * significant at *p* < 0.05. ns non-significant.

**Table 4:** The effect of growth regulator combinations on the regeneration efficiency of the AC-derived calli.

Induction Media	N_6_NDK				
	Number of Regenerated Plantlets/100 Anthers				
	Plant Regeneration Media				
Genotype	mMSNBK1	MSNBK3	MSNBKZ1	MSNBKZ2	Mean of Media
‘Co 39’	18.4 a A	14.4 a A	5.2 b A	6.0 b A	11.000
‘PSB RC 94’	14.4 a AB	13.6 a A	0.8 b AB	1.2 b B	7.500
‘IRRI 147’	10.0 a B	4.8 b B	0.0 c B	0.0 c B	3.700
‘Mangala’	4.1 a C	5.9 a B	1.6 a AB	2.1 a AB	3.425
Mean of genotypes	11.725	9.675	1.900	2.325	6.406
	**Number of Albinos/100 anthers**				
	**Plant Regeneration Media**				
**Genotype**	**mMSNBK1**	**MSNBK3**	**MSNBKZ1**	**MSNBKZ2**	**Mean of Media**
‘Co 39’	18.0 a A	14.4 b A	4.8 c A	6.0 c A	10.800
‘PSB RC 94’	6.0 a B	8.4 a B	0.4 b B	0.4 b B	3.800
‘IRRI 147’	3.6 a BC	3.6 a C	0.0 b B	0.0 b B	1.800
‘Mangala’	0.5 a C	0.7 a C	0.4 a B	0.9 a B	0.625
Mean of Genotypes	7.025	6.775	1.400	1.825	4.256
	**Number of Green Plantlets/100 Anthers**				
	**Plant Regeneration Media**				
**Genotype**	**mMSNBK1**	**MSNBK3**	**MSNBKZ1**	**MSNBKZ2**	**Mean of Media**
‘Co 39’	0.4 a C	0.0 a B	0.4 a A	0.0 a A	0.200
‘PSB RC 94’	8.4 a A	5.2 b A	0.4 c A	0.8 c A	3.700
‘IRRI 147’	6.4 a AB	1.2 b B	0.0 b A	0.0 b A	1.900
‘Mangala’	3.6 ab B	5.2 a A	1.2 b A	1.2 b A	2.800
Mean of Genotypes	4.700	2.900	0.500	0.500	2.150

Note: Values followed by the same letters (a, b, c) are not significantly (*p* < 0.05) different for different growth regulator combinations within the genotype. Values followed by the same capital letters (A, B, C) are not significantly (*p* < 0.05) different for the genotypes using the same regeneration medium.

**Table 5:** The effect of plant regeneration media on the plant regeneration (green- and albino) of the calli induced using N_6_NDK induction medium.

Genotype	Reg. Media	Number of Transferred Calli	Number of Regenerated Green Plantlets	Number of Regenerated Albino Plantlets	Percentage of Green Plantlet Regeneration (%)	Percentage of Regeneration of Albino Plantlets (%)
‘Co 39’	mMSNBK1	475	1	45	0.21	9.47
	MSNBK3	475	0	36	0.00	7.58
	MSNBKZ1	475	1	11	0.21	2.32
	MSNBKZ2	475	0	15	0.00	3.16
‘PSB RC 94’	mMSNBK1	413	21	15	5.08	3.63
	MSNBK3	413	13	17	3.15	4.12
	MSNBKZ1	413	1	1	0.24	0.24
	MSNBKZ2	413	2	1	0.48	0.24
‘IRRI 147’	mMSNBK1	34	16	9	47.06	26.47
	MSNBK3	34	3	9	8.82	26.47
	MSNBKZ1	34	0	0	0.00	0.00
	MSNBKZ2	34	0	0	0.00	0.00
‘Mangala’	mMSNBK1	245	9	5	3.67	2.04
	MSNBK3	245	13	7	5.31	2.86
	MSNBKZ1	245	3	4	1.22	1.63
	MSNBKZ2	245	3	9	1.22	3.67
Summa	mMSNBK1	1167	47	74	4.03	6.34
	MSNBK3	1167	29	69	2.49	5.91
	MSNBKZ1	1167	5	16	0.43	1.37
	MSNBKZ2	1167	5	25	0.43	2.14
Total		4668	86	184	1.84	3.94

**Table 6:** The effect of combinations of growth regulators on the regeneration of the AC-derived calli induced using N_6_NDZ for induction of calli.

Induction Media	N_6_NDZ				
	Number of Regenerated Plantlets/100 Anthers				
	Plant Regeneration Media				
Genotype	mMSNBK1	MSNBK3	MSNBKZ1	MSNBKZ2	Mean of Media
‘Co 39’	13.6 a B	6.4 b C	1.6 bc AB	0.4 c B	5.50
‘PSB RC 94’	25.2 a A	21.6 a B	6.4 b AB	6.4 b AB	14.90
‘IR 64’	3.2 a C	4.0 a C	2.0 a AB	4.0 a AB	3.30
‘IRRI 147’	5.6 ab C	6.4 a C	0.0 b B	0.0 b B	3.00
‘Mangala’	23.2 b A	34.0 a A	7.6 c A	10.0 c A	18.70
Mean of Genotypes	14.16	14.48	3.52	4.16	9.08
	**Number of Albinos/100 Anthers**				
	**Plant Regeneration Media**				
**Genotype**	**mMSNBK1**	**MSNBK3**	**MSNBKZ1**	**MSNBKZ2**	**Mean of Media**
‘Co 39’	13.6 a B	6.4 ab B	1.6 b A	0.4 b B	5.50
‘PSB RC 94’	24.8 a A	20.8 a A	6.0 b A	6.4 b AB	14.50
‘IR 64’	3.2 a C	4.0 a B	2.0 a A	3.6 a AB	3.20
‘IRRI 147’	5.6 a C	6.4 a B	0.0 a A	0.0 a B	3.00
‘Mangala’	18.4 a AB	21.2 a A	4.8 b A	8.8 b A	13.30
Mean of Genotypes	13.12	11.76	2.88	3.84	7.90
	**Number of Green Plantlets/100 Anthers**				
	**Plant Regeneration Media**				
**Genotype**	**mMSNBK1**	**MSNBK3**	**MSNBKZ1**	**MSNBKZ2**	**Mean of Media**
‘Co 39’	0.0 a B	0.0 a B	0.0 a B	0.0 a A	0.00
‘PSB RC 94’	0.4 a B	0.8 a B	0.4 a B	0.0 a A	0.40
‘IR 64’	0.0 a B	0.0 a B	0.0 a B	0.4 a A	0.10
‘IRRI 147’	0.0 a B	0.0 a B	0.0 a B	0.0 a A	0.00
‘Mangala’	4.8 b A	12.8 a A	2.8 c A	1.2 c A	5.40
Mean of Genotypes	1.04	2.72	0.64	0.32	1.18

Note: Values followed by the same letters (a, b, c) are not significantly (*p* < 0.05) different for different growth regulator combinations within the genotype. Values followed by the same capital letter (A, B, C) is not significantly (*p* < 0.05) different for the genotypes using the same regeneration medium.

**Table 7:** The effect of plant regeneration media on the plantlet regeneration (green and albino) of the calli induced using N_6_NDZ induction medium.

Genotype	Reg. Media	Number of Transferred Calli	Number of Regenerated Green Plantlets	Number of Regenerated Albino Plantlets	Percentage of Green Plantlet Regeneration (%)	Percentage of Regeneration of Albino Plantlets (%)
‘Co 39’	mMSNBK1	546	0	33	0.00	6.04
	MSNBK3	546	0	15	0.00	2.75
	MSNBKZ1	546	0	3	0.00	0.55
	MSNBKZ2	546	0	1	0.00	0.18
‘PSB RC 94’	mMSNBK1	222	1	62	0.45	27.93
	MSNBK3	222	2	50	0.90	22.52
	MSNBKZ1	222	1	8	0.45	3.60
	MSNBKZ2	222	0	16	0.00	7.21
‘IR 64’	mMSNBK1	65	0	8	0.00	12.31
	MSNBK3	65	0	10	0.00	15.38
	MSNBKZ1	65	0	5	0.00	7.69
	MSNBKZ2	65	1	9	1.54	13.85
‘IRRI 147’	mMSNBK1	75	0	14	0.00	18.67
	MSNBK3	75	0	16	0.00	21.33
	MSNBKZ1	75	0	0	0.00	0.00
	MSNBKZ2	75	0	0	0.00	0.00
‘Mangala’	mMSNBK1	411	11	45	2.68	10.95
	MSNBK3	411	32	56	7.79	13.63
	MSNBKZ1	411	7	12	1.70	2.92
	MSNBKZ2	411	3	22	0.73	5.35
Summa	mMSNBK1	1319	12	162	0.91	12.28
	MSNBK3	1319	34	147	2.58	11.14
	MSNBKZ1	1319	8	28	0.61	2.12
	MSNBKZ2	1319	4	48	0.30	3.64
Total		5276	58	385	1.10	7.30

**Table 8:** Ploidy levels of AC—derived acclimatized plantlets based on flow cytometric analyses.

Genotype	Haploid (n)	Diploid (2n)	Tetraploid (4n)	Mixoploid (n-2n)	Number of Tested Acclimatized Plantlets
‘Co 39’	1	0	0	0	1
‘PSB RC 94’	13	13	0	0	26
‘IR 64’	1	0	0	0	1
‘IRRI 147’	0	15	0	0	15
‘Mangala’	33	27	2	1	63
Total Number (Percentage) of Plantlets with Different Ploidy Levels	48 (45.28%)	55 (51.89%)	2 (1.89%)	1 (0.94%)	106 (100%)

**Table 9:** Components of induction medium in rice AC.

Components of Medium (mg L^−1^)	N_6_NDK	N_6_NDZ	Ali-1
Macronutrients	N_6_	N_6_	N_6_
H_3_BO_3_	1.6	1.6	1.6
MnSO_4_ × 4H_2_O	4.4	4.4	4.4
ZnSO_4_ × 7H_2_O	1.85	1.85	1.5
KI	0.8	0.8	0.8
FeSO_4_ × 7H_2_O	27.85	27.85	27.85
Na_2_EDTA × 2H_2_O	37.25	37.25	37.25
Nicotinic acid	0.5	0.5	0.5
Thiamine HCl	1	1	1
Pyridoxine HCl	0.5	0.5	0.5
Myo-inositol	-	-	100
Sucrose	-	-	30,000
Maltose	40,000	40,000	30,000
Glycine	-	-	10
L-Proline	500	500	-
L-Glutamine	500	500	-
2,4-D	1	1	1
NAA	2.5	1	1
Kinetin	0.5	-	-
Zeatin	-	0.1	0.1
pH	5.8	5.8	5.8
Phytagel	2800	2800	2000

**Table 10:** Combinations of growth regulators in plant regeneration media of indica rice.

Regeneration Medium	mMSNBK1	MSNBK3	MSNBKZ1	MSNBKZ2
NAA (mg/L)	1	0.5	0.5	1
BAP (mg/L)	1	0.5	0.5	1
Kinetin (mg/L)	1	1.5	1.5	1
Zeatin (mg/L)	-	-	0.25	0.25

## Data Availability

All data used in this manuscript are presented in the manuscript.

## Abbreviations

2,4-D	2,4-Dichlorophenoxyacetic acid
AC	Anther culture
BAP	6-Benzylaminopurin
DH	Doubled haploid
NAA	1-Naphtylacetic acid
MS	Murashige and Skoog
